# A Polysaccharide Isolated from *Codonopsis pilosula* with Immunomodulation Effects Both In Vitro and In Vivo

**DOI:** 10.3390/molecules24203632

**Published:** 2019-10-09

**Authors:** Yuan-Feng Zou, Yan-Yun Zhang, Yu-Ping Fu, Kari Tvete Inngjerdingen, Berit Smestad Paulsen, Bin Feng, Zhong-Kai Zhu, Li-Xia Li, Ren-Yong Jia, Chao Huang, Xu Song, Cheng Lv, Gang Ye, Xiao-Xia Liang, Chang-Liang He, Li-Zi Yin, Zhong-Qiong Yin

**Affiliations:** 1 Natural Medicine Research Center, College of Veterinary Medicine, Sichuan Agricultural University, Chengdu 611130, China; 18684157526@163.com (Y.-Y.Z.); yupingfu424@163.com (Y.-P.F.); zhongkaizhu6@163.com (Z.-K.Z.); lilixia905@163.com (L.-X.L.); songx@sicau.edu.cn (X.S.); lvcheng1980@163.com (C.L.); yegang800206@163.com (G.Y.); liangxiaoxia@sicau.edu.cn (X.-X.L.); lorri190@126.com (C.-L.H.); yinlizi@hotmail.com (L.-Z.Y.); 2 Department of Pharmacy, Section Pharmaceutical Chemistry, University of Oslo, P.O. Box 1068 Blindern, 0316 Oslo, Norway; k.t.inngjerdingen@farmasi.uio.no (K.T.I.); b.s.paulsen@farmasi.uio.no (B.S.P.); 3 Animal Nutrition Institute, Sichuan Agricultural University, Chengdu 611130, China; fengbin@sicau.edu.cn; 4 Key Laboratory of Animal Disease and Human Health of Sichuan Province, College of Veterinary Medicine, Sichuan Agricultural University, Chengdu 611130, China; jiary@sicau.edu.cn (R.-Y.J.); huangchao@sicau.edu.cn (C.H.)

**Keywords:** *Codonopsis pilosula*, polysaccharide, Peyer’s patch, immunomodulation

## Abstract

In this study, an acidic polysaccharide from *Codonopsis pilosula* Nannf. var. *modesta* (Nannf.) L. T. Shen (WCP-I) and its main fragment, WCP-Ia, obtained after pectinase digestion, were structurally elucidated and found to consist of a rhamnogalacturonan I (RG-I) region containing both arabinogalactan type I (AG-I) and type II (AG-II) as sidechains. They both expressed immunomodulating activity against Peyer’s patch cells. Endo-1,4-β-galactanase degradation gave a decrease of interleukine 6 (IL-6) production compared with native WCP-I and WCP-Ia, but exo-α-l-arabinofuranosidase digestion showed no changes in activity. This demonstrated that the stimulation activity partly disappeared with removal of β-d-(1→4)-galactan chains, proving that the AG-I side chain plays an important role in immunoregulation activity. WCP-Ia had a better promotion effect than WCP-I in vivo, shown through an increased spleen index, higher concentrations of IL-6, transforming growth factor-β (TGF-β), and tumor necrosis factor-α (TNF-α) in serum, and a slight increment in the secretory immunoglobulin A (sIgA) and CD4^+^/CD8^+^ T lymphocyte ratio. These results suggest that β-d-(1→4)-galactan-containing chains in WCP-I play an essential role in the expression of immunomodulating activity. Combining all the results in this and previous studies, the intestinal immune system might be the target site of WCP-Ia.

## 1. Introduction

In recent years, several undigested natural polysaccharides have become of interest for use as healthcare products and natural drugs, as the majority of related studies have demonstrated their immune-modulation effects. This effect could occur through the intestinal microbiota and immune system, for example through targeting bacteria, immune cells, oxidant stress, or short-chain-fatty-acids [1]. Gut-associated lymphoid tissues (GALTs) are predominantly immune modulation nodes providing a bond between microbiota and host systemic immunity, causing a response at the mucosal interface. Furthermore, GALTs have vital features in the control of proliferation and composition of microbes [2]. In addition, Peyer’s patches are lymphoid follicles in GALTs, transporting luminal antigens, and are considered as immune sensors of the intestine to achieve contact between immune cells in the lymphoid follicles and the associated epithelium [3]. It has been shown that the immunomodulating effect in Peyer’s patches was related to polysaccharides with specific moieties such as β-d-(1→3,6)-galactan and β-d-(1→4)-galactan in expression of bioactivity [4,5].

Radix Codonopsis, traditionally used in Chinese medicine, is used as a replacement for *Panax ginseng* since it has the same pharmacological activities with lower price. One of its bioactive components is polysaccharide, which works as an immunological enhancer, microecological balancer, and tumor killer. All our previous experiments proved that the polysaccharides from *Codonopsis pilosula* Nannf. var. *modesta* (Nannf.) L. T. Shen (WCP), one of three species of *C. pilosula*, has activities with respect to intestinal immunity and microbiota immunomodulation, complement fixing, and prebiotic effects [6,7,8]. Particularly, the acidic fractions 50 WCP-II-I and 100 WCP-II-I, obtained using an accelerate solvent extraction (ASE) method with different temperatures (50 °C and 100 °C) and pre-extracted with two different concentrations of ethanol (50% and 96%), had these effects [6]. ASE is not an extraction method for wholesale industrial use, but provides repeatable extraction processes, and may be more preferable for analysis of content of low-molecular-weight components. Traditionally, water extraction was the main, conventional, energy-saving method for extraction of polysaccharides. The extraction method could be one of the determinants influencing underlying compositional and/or structural change, especially for the bioactive groups. For instance, various extraction conditions like different temperatures could lead to a different yield, monosaccharide composition (especially uronic acid), molecular weight, and bioactive effect, as well as physical changes in the size and shape of the polymer [9,10,11,12]. 

The aim of this paper was, based on our previous complement fixing study in vitro, to investigate immunomodulating effects of polysaccharides from *C. pilosula* Nannf. var. *modesta* (Nannf.) L. T. Shen (WCP) isolated through a completely different method. Then, the relationship between structure and intestinal immune modulation activity of the pectin WCP-I and its derivatives degraded by different enzymes could be studied through comprehensive evaluation both in vitro and in vivo.

## 2. Results

### 2.1. Structural Characterization of WCP-I and Its Enzymatic Derivatives

**Figure 1:**
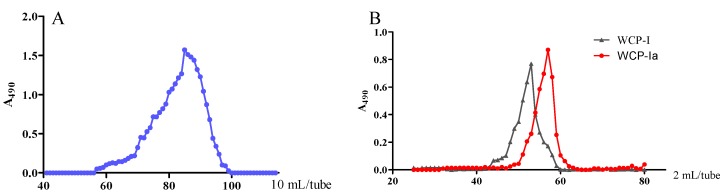
Elution profiles. (**A**) elution profile of WCP by diethylaminoethyl (DEAE)-Sepharose Fast Flow; (**B**) size exclusion chromatography elution profile of fraction WCP-I (black) and its degradation product by pectinase (red).

One acidic fraction isolated from *C. pilosula*, WCP-I (Figure 1A), accounts for 19% (*w*/*w*) of WCP. The monosaccharides and linkage elucidation of this fraction are shown in Table 1 and Table 2, respectively. The WCP-I consists mainly of GalA, Gal, Rha, and Ara, followed by Man and Glc (<1%). According to previous studies, all its glycoside types demonstrated that it is a typical pectin, with a branched rhamnogalacturonan type I (RG-I) backbone (hairy region) and long homogalacturonan chains (HGA), identified by a lower ratio of Rha (1→2,4 and 1→2 linked) to GalA (1→4 linked) [6,13]. As shown in Table 2, the presence of both 1→3,6 and 1→4 linked Gal*p* indicated the presence of AG-II and AG-I regions in WCP-I, as found by Inngjerdingen et al. [13]. There were linear arabinans linked at positions 2 or 3, or single Ara*f* connected on Gal*p* (AG-II), but this did not occur at position 5 since no 1→5 linked Ara*f* was detected.

The treatment with endo-α-(1-4)-polygalacturonase (pectinase) led to a decrease of GalA, especially 1→4 linked GalA, and a disappearance in WCP-Ia of terminal GalA, which meant that part of the homogalacturonan (HGA ) chains in WCP-I was probably degraded by pectinase. The elution profile given in Figure 1B shows a substantial reduction of molecular size after pectinase degradation. 

After the degradation with exo-α-l-arabinofuranosidase, the content of Ara of WCP-Ib decreased, accompanied by a 4.8% increasement of Gal (Table 1). The enzymatic product of exo-α-l-arabinofuranosidase treatment was followed by digestion with endo-1,4-β-galactanase (WCP-Ic). As can be seen in Table 1, about 4.2% of Gal was released, which partly destroyed the AG-I region (data not shown). 

It is important to mention that compared with our previous research in 2014 [6], a different extraction method altered the structure of this bioactive pectic polymer. The polymer was then identified as having linear arabinans with both 1→5 and 1→3,5 linked Ara*f* (50-WCP-II-I and 100 WCP-II-I), and consisted of 1→4 linked Glc*p*, and 1→4 linked GlcA, which were not found in WCP-I of the present study (Table 2).

### 2.2. The Intestinal Immunological Activity In Vitro

**Figure 2:**
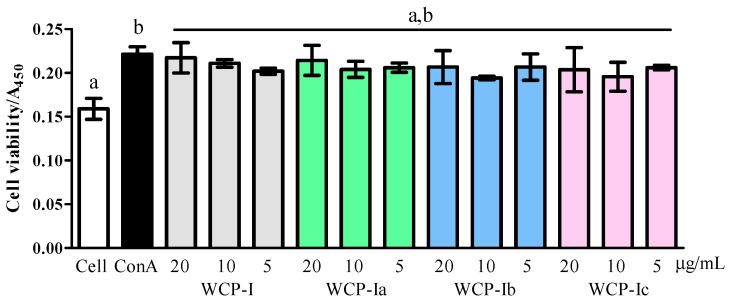
Cell viability of Peyer’s patch cells. Cell viability was expressed as the absorption value in 450 nm according to the manufacturer’s instructions for CCK-8 kits. The different marked letters indicate a significant difference, *p* < 0.05.

**Figure 3:**
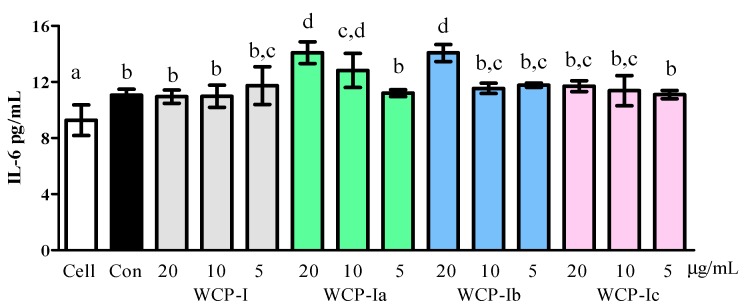
IL-6 secretion in the supernatant culture of Peyer’s patch cells. The different marked letters indicate a significant difference, *p* < 0.05.

The intestinal epithelium provides a huge surface area composed of a single layer of intestinal epithelial cells, an interface for intestinal microbiota and local immune cells. The macromolecular polysaccharides were considered to have an immunity enhancement effect, probably through the direct reaction with intestinal epithelial cells in GALTs like Peyer’s patch cells, and different structural polysaccharides will show functional variation in the intestine [14]. These cells grew significantly after the addition of concanavalin (ConA) (Figure 2), and the concentration of interleukin-6 (IL-6) was significantly higher in groups with added ConA than in the control group (Figure 3, *p* < 0.05). Those groups supplemented with polymers did not change compared to the control group (containing ConA). However, as can be seen in Figure 3, WCP-Ia (20 and 10 μg/mL) and WCP-Ib presented an IL-6 stimulation effect in the supernatant of cell culture compared with the negative effect of native WCP-I and the WCP-Ic (*p* < 0.05).

### 2.3. The Intestinal Immunological Activity In Vivo

#### 2.3.1. Systematic Immunity Modulation

**Figure 4:**
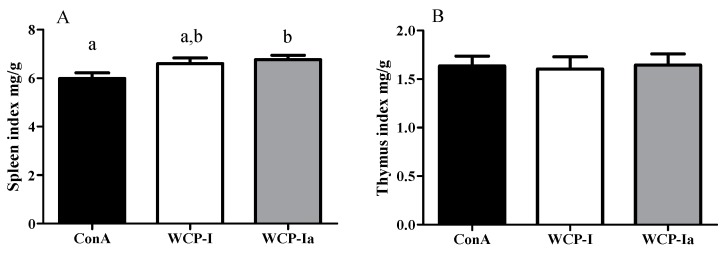
Immune organ indexes of C3H/HeJ mice treated with WCP-I and WCP-Ia. The different marked letters indicate a significant difference, *p* < 0.05.

**Figure 5:**
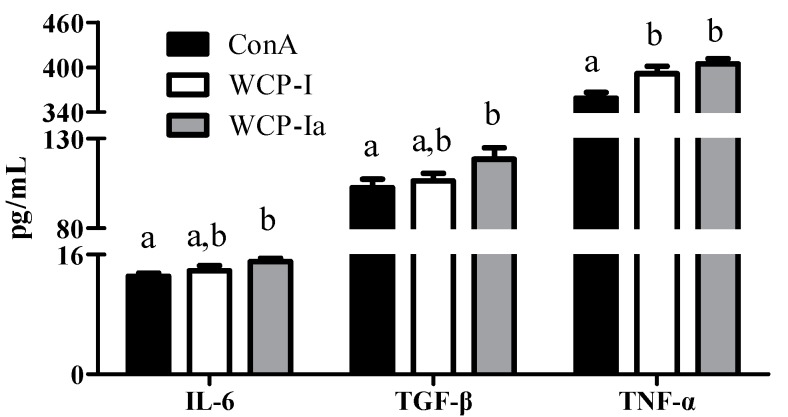
Cytokine secretion in serum of C3H/HeJ mice treated with WCP-I and WCP-Ia. The different marked letters indicate a significant difference, *p* < 0.05.

Considering better applications and industrial products in the future, we analyzed the product with better activity and a higher yield (WCP-Ia) on C3H/HeJ mice, which would explain the primary active immunomodulating mechanism and structural relationship. As shown in Figure 4, the spleen index was increased by WCP-I and WCP-Ia (*p* < 0.05), but without significant changes in the thymus index, indicating a potential immunity enhancement of these two samples. At the same time, the WCP-Ia elevated the production of IL-6, transforming growth factor-β (TGF-β), and tumor necrosis factor-α (TNF-α) in serum significantly compared with the ConA group (*p* < 0.05), as shown in Figure 5. Moreover, it revealed a better promotion trend than WCP-I, though without significant consequences (*p* > 0.05), in accordance with the results in vitro. All these results proved WCP-Ia a systematic immunity modulator and functions better than WCP-I.

#### 2.3.2. The Intestinal Immunity Modulating Effect

**Figure 6:**
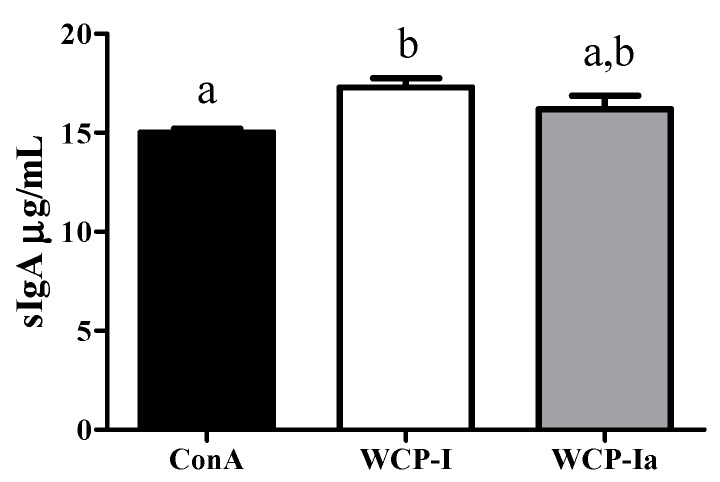
sIgA secretion of C3H/HeJ mice treated with WCP-I and WCP-Ia. All the values show sIgA concentration in ileum tissues, and the different marked letters indicate a significant difference, *p* < 0.05.

Several scientists have been working on the immune-regulation of natural polysaccharides. Based on its undigested feature in upper gastrointestinal tract, we have now been focusing on the intestinal mucosal immunity, expressed as the connection bridge between epithelium and polysaccharides. Firstly, levels of secretory immunoglobulin A (sIgA), the first defense line in the intestinal mucosal immunity, rose after the WCP-I administration compared with the ConA group (*p* < 0.05), representing its underlying modulation effect on intestinal mucous membrane (Figure 6). However, the mice treated with WCP-Ia showed a slight enhancement in sIgA levels (*p* > 0.05) compared with the ConA group, but with no significant difference between WCP-I and WCP-Ia. 

The production of sIgA is dependent on Peyer’s patch M-cells and processing by antigen-presenting cells such as dendritic cells (DCs), T-cell activation, or ultimately B-cell class switch recombination in GALTs [15]. To clarify the underlying reason and the exact lymphocytes changing in intestinal mucosa by these polysaccharides with distinctive structural features, the Peyer’s patch cells were stained with various fluorescent antigens and subjected to flow cytometry (results given in Table 3). The proliferation response of T and B lymphocytes in Peyer’s patch did not change with WCP-I or WCP-Ia, probably indicating that there was little effect on lymphocyte proliferation. The differentiation of T lymphocytes in WCP-I and WCP-Ia groups divided into CD4^+^ T cells revealed increased CD4^+^ cells (*p* > 0.05) and an immobile CD8^+^ cell count, with a significant CD4^+^/CD8^+^ ratio boost in helper T cell (Th cell) populations, especially WCP-Ia (*p* < 0.05). 

## 3. Discussion

Polysaccharides from herbal medicine have attracted attention and play various and vital roles in many biological processes due to their structural diversity, low toxicity, and distinct biological activities, especially the regulation of the immune system [16]. A multitude of plant polysaccharides have showed immunity enhancement effect in vitro, like complement fixing or lymphocyte stimulation, which are thought be the result of specific structures or groups such as 1→3,6 branched galactosyl residues, arabinans, and the most dominant core, the rhamnogalacturonan [17]. In our studies, the WCP-Ia and WCP-Ib fractions showed a more potent immunomodulating effects than the native WCP-I, which is in accordance with our previous work on complement fixing activity [6]. The potential function of immunocompetent stimulation in the Peyer’s patches of the mucosal immune system of the isolated fractions was indicated here. Some of the relations between their structure and the activity are discussed as follows.

Firstly, the presence of neutral residues of Ara*f* units was not necessary, as demonstrated by a retained bioactivity of WCP-Ib. Choi et al. considered that the intestinal immune system-modulating activity of the aerial parts from *Astragalus membranaceus* was induced by a polygalacturonan moiety with neutral sugars such as arabinose and galactose [18], and Diallo et al. showed a reduced complement fixing activity after the removal of terminal Ara*f* [19]. Le Normand also found a RG-I domain polymer ramified with highly-branched arabinans from spruce bark with immunomodulating activities [20]. However, not all the natural polysaccharides displayed this effect. For instance, Inngjerdingen et al. [13] and Nergard et al. [21] indicated that partial removal of Ara*f* residues did not have effect on the complement fixing activity and the intestinal immune stimulating test after enzymatic hydrolysis, and Sakai suggested that polysaccharides from *Saccharum officinarum* digested with mixed enzymes of exo-β-d-1,3-galactanase and endo-β-d-1,4-galactanase, in the presence of exo-α-l-arabinofuranosidase, did not change the Peyer’s patch-immunomodulating activity [22]. All these results are similar to what we found. Still, a clear relationship of the biological importance of branched Ara*f* units remains uncertain; they were not essential, at least in the polysaccharide from *C. pilosula*. It may also be so that slight differences in the structural part of the polysaccharides influence the role of the arabinose units, thus being important in some structures and not important in others. 

Secondly, the majority of enzymatic studies have paid greater attention to the β-d-(1→3,6)-galactan structure, and have identified it as the main domain in side chains of RG-I. The β-d-(1→4)-galactan chains were first confirmed in 2011, contributing to enhancing the activity of Peyer’s patch cells for the immunomodulating activity of polysaccharides [5] and not only β-d-(1→3,6)-galactan chains. Similarly, its crucial role was shown in our study because removal of the β-d-(1→4)-galactan chains of WCP-Ic had a negative effect on IL-6 secretion. 

The polysaccharides from *C. pilosula* in our previous studies have exerted immunological modulation activity both in vitro [6] and in vivo [8]. Combining all results in vitro and the secretion of cytokines, it was demonstrated that WCP-Ia generally displayed a more effective immunoregulation function than WCP-I. Thus, we speculated if this was rooted in the Peyer’s patches, whose cytokine production could be enhanced by various natural polysaccharides, with most in a dose-dependent manner such as from *Hordeum vulgare* L. [23], *Astragalus membranaceus* [18], *Entada africana* [19], and *Saccharum officinarum* [22]. It could also account for the binding reaction on carbohydrate recognition domain (CRD), such as the modified pectin with polygalacturonic acid and RG-I regions that cleaved and de-esterified the HG backbone [24].

Thus, the intestinal immunological activity was determined, and the sIgA in ileum tissues was increased in the WCP-I group, in accordance with our previous conclusion. The WCP-Ia here showed a negative effect, but could activate directly through endoplasmic reticulum in epithelial cells or change the microbiota composition [25]. This could also be due to T lymphocyte differentiation rather than the B cell proliferation, as shown in Table 3. At the same time, the T follicular helper (Tfh) cells in Peyer’s patches supported affinity maturation of B cells in germinal centers (GCs) [26] and differentiation of B cells to IgA-secreting plasma cells, indicating that the un-activated status of Tfh cell could be another cause. Immune regulation relies mainly on helper T cells (CD4^+^ T) and cytotoxic T cells (CD8^+^ T) and its ratio, which is considered as a vital index in immunocompetence evaluation. Many natural polysaccharides are able to enhance T lymphocyte proliferation and increased the levels of CD4^+^ and CD8^+^ T cells [27,28]. There was no difference with respect to the conclusion of Georgiev et al. that the approach of polysaccharides expressing activity against the Peyer’ patch mediated-bone marrow cell proliferation was through phagocytic leukocytes and T-lymphocytes [29]. Some natural polysaccharides from *Astragalus membranaceus*, *Panax ginseng*, and *Ganoderma lucidum* for example improved the percentage of activated Th cells in the Peyer’ patch cells, promoting CD4^+^ T cell growth and inhibiting CD8^+^ T cell [30,31,32]. In addition, the lymphocyte differentiation was proven by the cytokines in serum, since the modified pectin could potentially facilitate CD4^+^ T lymphocyte differentiation towards Treg cells (induced by TGF-β independently and secreting TGF-β at the same time), or could differentiate into Th17 cells under the co-induction of TGF-β and IL-6 and then participate in inflammatory response. At times the differentiation of mucosal dendritic cells (DCs) could be driven by the release of TGF-β in intestinal epithelial cells, which could be activated by local factors like proteases [33,34]. TNF-α, mainly produced by macrophages and Th1 subset of CD4^+^ T cells, was one of the cytokines effected sharply by WCP-Ia (Figure 3), as proven by our flow cytometric analysis again.

As various studies of pectin polysaccharide activities on immune function have shown, the RG domain is essential for its immunoregulation activity, with its branched arabinans and galactan chains both as β-d-(1→3,6)- and β-d-(1→4) [35]. WCP-Ia, structurally optimized by pectinase with more intensive AG region, exhibited a better immunocompetence, which was identified first by experiments in vitro by complement fixing activity [6] and cytokine secretion of Peyer’s patch cells, and then verified in vivo through promoting T lymphocyte cell differentiation towards CD4^+^ T cells of Peyer’s patches. Combining our previous results in vivo [8] and the index changes in this study, the intestinal immune system might be a target active site of this polymer.

## 4. Materials and Methods

### 4.1. Materials and Chemicals

The roots of *C. pilosula* Nannf. var. *modesta* L. T. Shen were collected in October 2017 from Jiuzhaigou County (Tibetan Qiang Autonomous Prefecture of Ngawa, China), and identified by Yuan-Feng Zou, College of Veterinary Medicine, Sichuan Agricultural University. The roots were dried and pulverized to a fine powder. The endo-α-(1-4)-polygalacturonase (pectinase, P2611) from *Aspergilus aculeatus* was purchased from Sigma-Aldrich, Saint Louis, USA; the exo-α-l-arabinofuranosidase (E-ABFCT) from *Clostridium thermocellum*, and endo-1,4-β-d-galactanase (E-EGALN) from *Aspergillus niger* were purchased from Megazyme, Ireland. All the mentioned ELISA kits were purchased from Enzyme-linked Biotechnology Co., Ltd., (Shanghai, China). The DNS reagent and all other chemicals such as phenol, sulfuric acid, ethanol, etc., were of analytical grade, obtained from the Chengdu Kelong chemical factory (Chengdu, China).

### 4.2. The Extraction and Purification of Acidic Polysaccharides

After being dried at 40 °C, the powered materials were extracted using 96% ethanol and boiling water successively, and then the crude polysaccharides were obtained by ethanol-precipitation described as our previous work [8]. This lyophilized polysaccharide powder was dissolved and dialyzed at cut-off 3500 Da, and then lyophilized again and named WCP (water extracted-polysaccharides from *C. pilosula* Nannf. var. *modesta* (Nannf.) L. T. Shen). Continuously, the aqueous sample was filtered through a 0.45-μm filter membrane (400~500 mg, 20 mL) and applied to an anion exchange column (GE Healthcare Bio-Sciences, Uppsala, Sweden) packed with DEAE-Sepharose Fast Flow (Beijing Rui Da Heng Hui Science Technology Development Co., Ltd., Beijing, China). The acidic fractions were eluted with a linear NaCl gradient in water (0~1.5 M) at 2 mL/min after eluting the neutral fractions with distilled water. The carbohydrate elution profiles were monitored using the phenol-sulfuric acid assay [36], and the relevant fraction was pooled into one. Then, the collected fraction was concentrated and dialyzed to remove NaCl, lyophilized, and named WCP-I.

### 4.3. Enzymatic Degradation

#### 4.3.1. Endo-α-(1-4)-Polygalacturonase Degradation

The WCP-I was first digested by endo-α-(1-4)-polygalacturonase (pectinase) to produce “hairy” regions, performed as in previous studies [6]. The end of degradation was identified by DNS reagent, and the degraded fraction was further fractionated by the Hiload^TM^26/60 Superdex^TM^200 pre-grade column (GE Healthcare) with 50 mM NaCl at 0.5 mL/min, 2 mL/tube, linked with NGC™10 medium-high pressure chromatography system (Bio-Rad). The carbohydrate elution profile was determined with the phenol-sulphuric acid method. 

#### 4.3.2. Exo-α-l-Arabinofuranosidase and Endo-1,4-β-Galactanasedegradation

The enzymatic degradation of exo-α-l-arabinofuranosidase and endo-1,4-β-galactanase was based on Nergard et al. [21] with minor modifications. Firstly, WCP-Ia (10~12 mg) in 2 mL acetate buffer (pH = 4.2, 30 mM) was digested with arabinofuranosidase (50 μL) in glass tubes previously washed with concentrated hydrochloric acid, incubated under shaking at 37 °C for 72 h, and then inactivated with boiling water. The mixed solution was purified using PD-10 column with Sephadex G-25 (GE Healthcare) according to the instructions, and lyophilized and named WCP-Ib. Then, WCP-Ib (10~12 mg) 2 mL acetate buffer (pH = 4.2, 30 mM) was digested with 50 μL endo-1,4-β-galactanase, and reacted for 48 h at 37 °C. The mixed solution was purified using PD-10 column, and lyophilized, named WCP-Ic.

### 4.4. Determination of Monosaccharide Composition and Glycosidic Linkage

The monosaccharides component was confirmed by gas chromatography (GC) after converting samples to their methyl glycosides by trimethylsilane (TMS) after methanolysis on the basis of the methods from Barsett, Paulsen, Habte (1992) [37] and Chambers & Clamp (1971) [38]. The glycoside linkage elucidation was performed by methylation after reduction with NaBD_4_, methylation, hydrolysis, reduction with sodium borodeuteride and acetylation [39]. The data were characterized by interpretation of the mass spectra and the retention times in relation to the standard sugar derivatives, as described previously [6].

### 4.5. The Intestinal Immunological Activity In Vitro

C3H/HeJ mice (female) were purchased from Beijing Vital River Laboratory Animal Technology Co., Ltd., Beijing, P.R. China. All animal procedures were reviewed and approved by the Animal Care and Use Committee of Sichuan Agricultural University.

The Peyer’s patch cells in vitro and in vivo were prepared from C3H/HeJ mice, as described previously [4,40], and were suspended with basic medium containing ConA (final concentration of 5 μg/mL) to 1 × 10^7^ cells/mL. For in vitro experiments, 180 μL of cell suspension were divided into 14 groups on 96-well plate (Table 4), and then 20 μL of aqueous solution of different samples or basic medium were added and co-cultured for 3 days in a humidified 37 °C atmosphere of 5% CO_2_/95% air. The supernatant (100 μL) was collected after centrifuging at 1500 rpm for 10 min, then 10 μL of CCK-8 was added to each well, and measured by Multiscan Spectrum (Bio-Rad) at 450 nm after continuous culture for 1 h. The modulatory activity of the intestinal immune system was performed using a population of live cells (fluorescence intensity) and comparison with the ConA group. The IL-6 concentration in supernatant was determined using ELISA kits. The groups were designed with six repetitions each.

### 4.6. The Intestinal Immunological Activity In Vivo

#### 4.6.1. Animal and Experimental Design

All animal procedures were reviewed under ethic approval (No. 2018-1210) by the Animal Care and Use Committee of Sichuan Agricultural University. The C3H/HeJ mice were maintained in a specific pathogen-free environment, with a 12 h light–dark cycle and 22 ± 1 °C room temperature, and provided with water and mouse chow ad libitum. After acclimatization under free access for 7 days, all these mice were randomly divided into three groups: the WCP-I (100 mg/kg/day, 0.1 mL/10 g bodyweight, the same below), WCP-Ia, and control groups (normal saline, 0.1 mL/10 g bodyweight). There were 10 mice in each group, and they were administrated with samples or saline for 10 days. Twenty-four hours after the last drug administration the animals were weighed and then sacrificed by decapitation. The spleen and thymus were isolated from the animals, and were weighed immediately to calculate the index according to the following formula: index (mg/g) = (weight of organ/body weight). The blood, ileum, and Peyer’s patches were obtained from all mice for the following studies.

#### 4.6.2. The Secretion of Cytokines in Serum and sIgA in Ileum

After the last administration for 24 h, the eyeball blood was collected and fully coagulated, and the serum was obtained after precipitation of 15 min, at 4 °C, 4000 rpm. The concentration of cytokines secreted in serum of mice, including IL-6, TGF-β, and TNF-α were detected by using mice ELISA kits according to the instructions. The content of sIgA in ileum was determined as procedures in our previous study [8].

#### 4.6.3. The Lymphocyte Differentiation in Peyer’s Patches by Flow Cytometry

The lymphocytes were isolated as mentioned in Section 4.5. After counting and washing with fresh sterile PBS (with 5% FBS, 500× *g*, 5 min) three times, the pelleted cells were re-suspended to 1 × 10^7^ cell/mL in PBS. The cell samples were divided into two tubes, 100 μL each, and antigens were added: PE/Cy7-anti-mouse CD3 (0.5 μg, the same below), FITC-anti-mouse CD4, PE-anti-mouse CD8a, and FITC-anti-mouse CD19, respectively (all Biolegend, San Diego, CA, USA) [41]. After mixing and incubation in dark at 4 °C for 30 min, the cells were washed with 900 μL cold-PBS twice, centrifuged (500× *g*, 5 min), and re-suspended with 300 μL PBS. The stained cells were analyzed with BD FACS Verse flow cytometer with FACSuite software (Becton Dickinson). Lymphocyte populations were determined as the percentages of T cells (CD3^+^, CD19^−^) and B cells (CD3^−^, CD19^+^) among leukocytes. Subpopulations of helper T cells and cytotoxic T cells are presented as the percentage of CD4^+^CD8^−^ and CD4^−^CD8^+^ cells among CD3^+^-expressing cells. 

### 4.7. Statistics

All results were expressed as the mean ± S.D., and the differences between groups were tested for statistical significance by ANOVA test and Duncan test. A value of *p* < 0.05 was considered to have statistical significance.

## 5. Conclusions

It has been reported that the immunological effects of several natural polysaccharides can be related to their structural features. A pectic polysaccharide was isolated from *Codonopsis pilosula* Nannf. var. *modesta* (Nannf.) L. T. Shen using boiling water, and was identified as having RG backbone and AG-I/-II structure, showing a different structure compared to previous acidic fractions extracted using ASE extraction. The enzymatic degradation analysis demonstrated that this polymer possesses β-d-(1→4)-galactan chains that contributed to its immunocompetence, promoted by the secretion of IL-6 in vitro and not the branched Ara*f* units. With both high yield and activity of the fraction, the pectinase-modified WCP-Ia exhibited an immunomodulation effect in C3H/HeJ mice, probably through intestinal Peyer’s patches, as shown by experiments in vitro and in vivo.

## Figures and Tables

**Table 1:** Monosaccharide compositions (%) of WCP-I and its enzymatic degradation products.

Samples	WCP-I	WCP-Ia	WCP-Ib	WCP-Ic
Arabinose (Ara)	5.5	13.0	10.1	11.0
Rhamnose (Rha)	6.4	10.6	11.1	12.6
Mannose (Man)	0.7	0.8	0.5	0.6
Galactose (Gal)	17.6	37.5	42.3	38.1
Glucose (Glc)	0.2	0.3	0.6	0.7
Galacturonic acid (GalA)	69.6	37.8	35.4	37.0

**Table 2:** Linkage elucidation of WCP-I and its pectinase degradation product WCP-Ia.

		WCP-I	WCP-Ia
Ara	T	2.8	6.7
	1→2	1.3	2.9
	1→3	1.4	3.3
Rha	1→2	3.3	5.5
	1→2,4	3.1	5.1
Gal	T	1.4	4.3
	1→4	4.5	9.2
	1→3	5.9	12
	1→6	3.1	6.3
	1→3,6	2.8	5.6
GalA	T	1.7	N.D ^1^
	1→4	67.9	37.8

^1^ N.D., not detected.

**Table 3:** Peyer’s patch cell subsets detected by flow cytometry.

Groups	T Cells (%)	B Cells (%)	CD4 (%)	CD8a (%)	CD4/CD8a
ConA	67.85 ± 0.35	83.70 ± 2.98	72.55 ± 1.77	95.00 ± 0.71	0.76 ± 0.01 ^a^
WCP-I	64.75 ± 3.18	80.43 ± 4.05	76.40 ± 0.14	95.60 ± 1.41	0.80 ± 0.01 ^ab^
WCP-Ia	67.30 ± 1.27	78.90 ± 2.63	76.90 ± 1.84	95.05 ± 0.21	0.81 ± 0.02 ^b^

Different marked letters indicate a significant difference, *p* < 0.05.

**Table 4:** Group design of cell culture.

Groups	T Cells (%)	ConA	Final Sample Concentration/(μg/mL)
Cell	Cell control	-	-
ConA	Control group	+	-
WCP-I	WCP-I	+	20
		+	10
		+	5
WCP-Ia	Different dosage of the pectinase digested product of WCP-I	+	20
		+	10
		+	5
WCP-Ib	Different dosage of the exo-l-arabinofuranosidase digested product of WCP-Ia	+	20
		+	10
		+	5
WCP-Ic	Different dosage of the digested product of endo-1,4-β-galactanase WCP-Ib	+	20
		+	10
		+	5
